# Case Report: A Squamous Cell Lung Carcinoma Patient Who Responded to Neoadjuvant Immunochemotherapy but Died From Anastomosis Leakage or/and irAEs: Immune Microenvironment and Genomic Features Changes

**DOI:** 10.3389/fonc.2021.674328

**Published:** 2021-07-22

**Authors:** Liping Hao, Ying Hu, Jianjun Hu, Yang Liu, Beibei Mao, Huan Chen, Xiaoli Gong, Di Wang, Lin Wang, Dong Wang

**Affiliations:** ^1^ Internal Medicine-Oncology, Cancer Centre of Jinling Hospital, Nanjing, China; ^2^ Center of Integrative Medicine, Beijing Ditan Hospital, Capital Medical University, Beijing, China; ^3^ Genecast Biotechnology Co., Ltd, Jiangsu, China; ^4^ Department of Thoracic Surgery, Affiliated Taikang Xianlin Drum Tower Hospital, Medical School of Nanjing University, Nanjing, China

**Keywords:** squamous cell lung carcinoma, immune-related adverse events, immune microenvironment, circulating cytokines, neoadjuvant and adjuvant immunochemotherapy

## Abstract

Clinical trials indicated that PD-1/PD-L1 inhibitors significantly improve the survival rate of patients with advanced non-small cell lung cancer (NSCLC) and induce immune-related adverse events (irAEs). Thus, the molecular and immune characteristics during PD-1/PD-L1 inhibitor therapy are worth investigating further. We report the case of a 62-year-old male patient diagnosed with stage IIIA squamous cell lung carcinoma (SQCC) who responded to neoadjuvant and adjuvant nivolumab combined chemotherapy but died from anastomosis leakage or/and irAEs. In the pretreatment tumor biopsy, PD-L1 expression was negative and a few T cells, NK cells, and macrophages had infiltrated the tumor. Wild-type *EGFR*/*STK11*, mutant *TP53*, microsatellite stability, and low tumor mutational burden were also found at baseline. After neoadjuvant immunochemotherapy, the tumor was significantly reduced, PD-L1 expression levels were increased by 50%, and more CD8^+^ and CD8^+^ PD-1^+^ T cells had infiltrated the resected tumor tissue. Immune-related lung injury occurred during adjuvant immunochemotherapy, and serum levels of C-reactive protein, IL-13, IL-4, eotaxin, VEGF-A, IL-8, and IFN-gamma were increased. This case demonstrates a squamous cell lung carcinoma patient who responded to neoadjuvant immunochemotherapy that reshaped the tumor immune environment from “cold” to “hot.” Unfortunately, the patient eventually died from anastomosis leakage or/and irAEs during adjuvant immunochemotherapy.

## Introduction

In the past few decades, clinical trials and studies have shown that PD-1/PD-L1 inhibitors significantly improve the survival rate of patients with advanced non-small cell lung cancer (NSCLC) (1–6). The National Comprehensive Cancer Network (NCCN) guidelines recommend PD-1/PD-L1 inhibitors as first-line therapy for advanced NSCLC (7). A pilot study published in the New England Journal of Medicine shed new light on neoadjuvant PD-1 blockade (nivolumab) in resectable early-stage NSCLC. This study found that in 45% of resected tumors, neoadjuvant nivolumab therapy induced a major pathological response (MPR) (8). Forty-six patients with stage IIIA lung cancer were recruited into the NADIM study. Before operation, patients received three cycles of nivolumab combined with carboplatin and paclitaxel therapy. The results showed that the MPR rate reached an unprecedented 85.36%, and the pathologic complete response (pCR) rate was 71.4% (9). In contrast, it was reported that immune-related adverse events (irAEs) were observed in 69 of 134 patients with advanced NSCLC who received nivolumab therapy. This study also revealed that irAEs were positively associated with survival outcomes (10). The molecular and immune characteristics of NSCLC patients who respond to neoadjuvant immunochemotherapy and suffer from irAEs require further study to facilitate overall patient management. Here, we present a case of a squamous cell lung carcinoma patient who had pathological regression in response to neoadjuvant and adjuvant PD-1 blockade combined with chemotherapy but died from irAEs; the molecular and immune characteristics before and after treatment and during the irAEs were examined.

## Case Presentation

**Figure 1:**
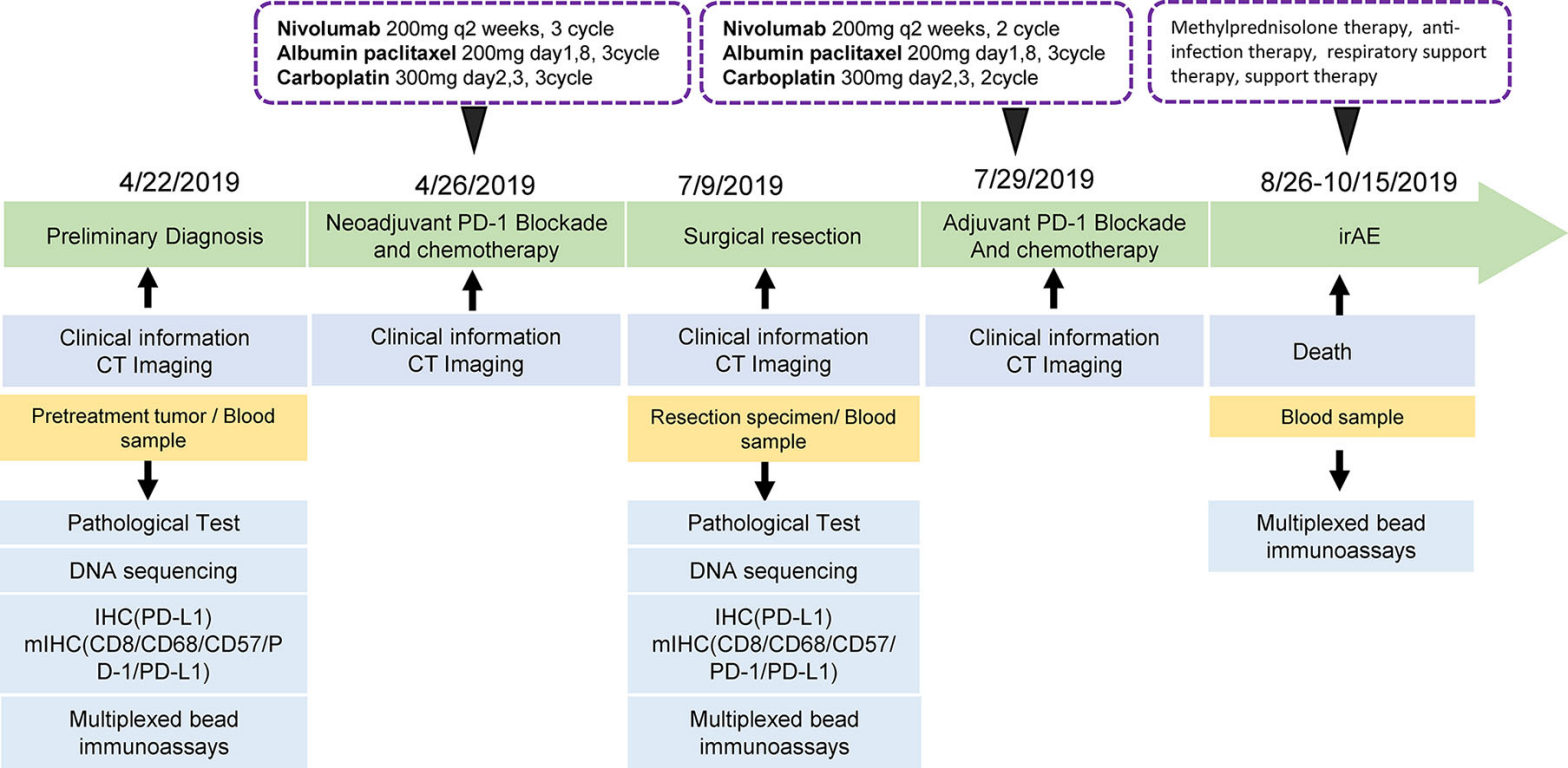
The timeline of the patient’s treatment course.

**Figure 2:**
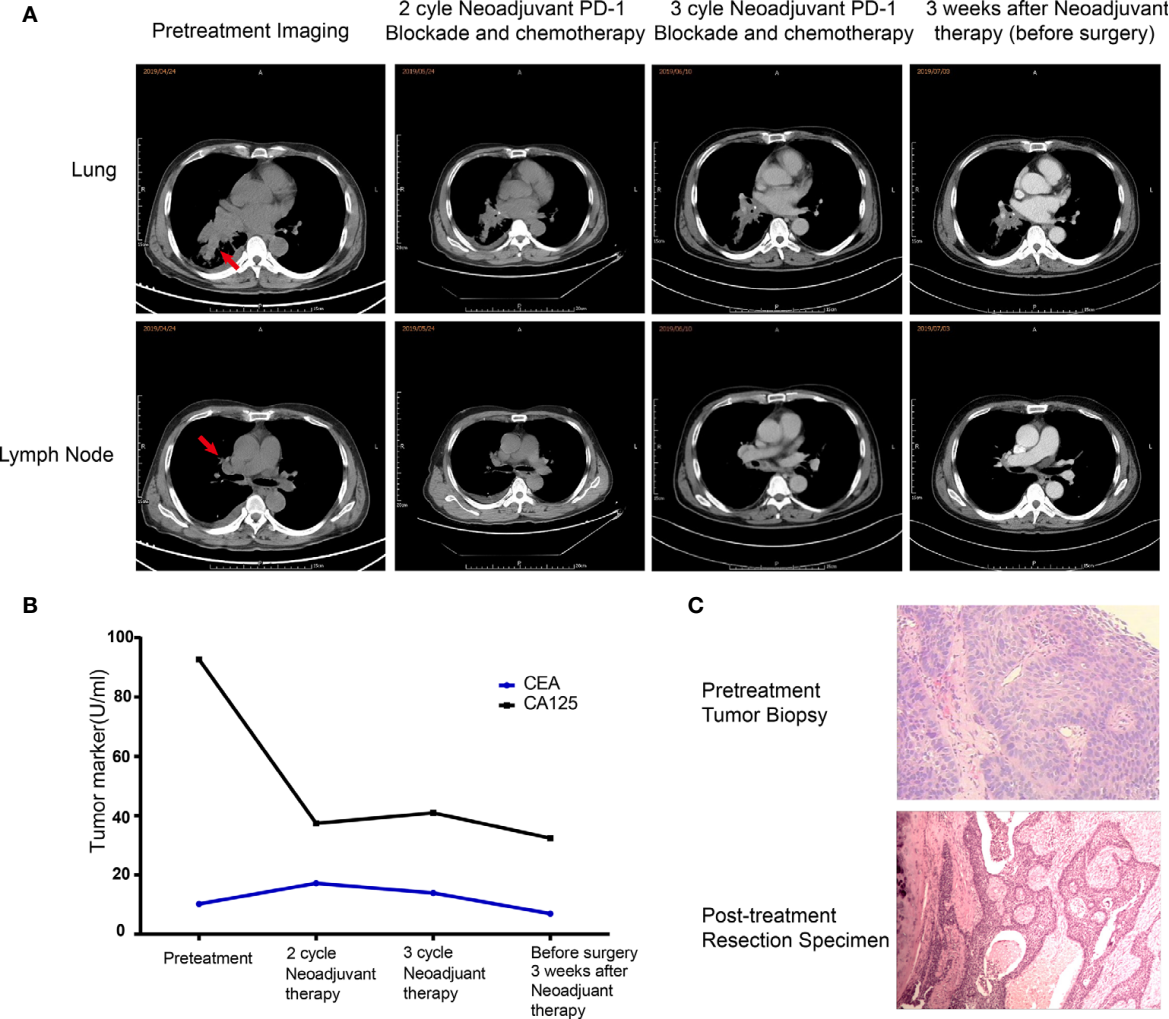
Changes in clinical indexes before and after neoadjuvant immunochemotherapy. **(A)** Representative CT images during neoadjuvant immunochemotherapy. **(B)** Carcinoembryonic antigen test results during neoadjuvant immunochemotherapy. **(C)** Histological results of the pretreatment tumor biopsy (hematoxylin and eosin (HE) staining, magnification ×200) and surgically resected tissue (HE, magnification ×100).

**Figure 3:**
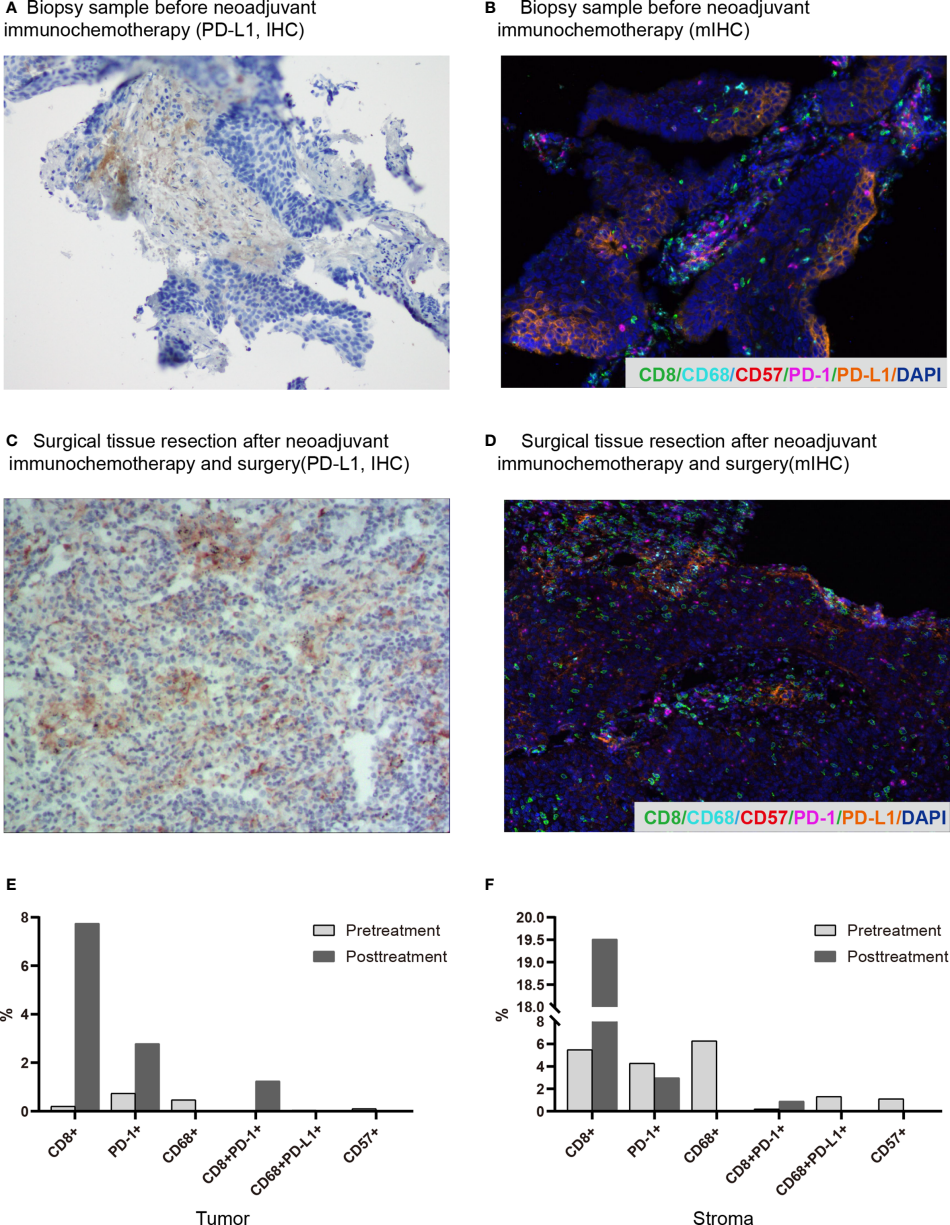
Changes in the immune microenvironment before and after neoadjuvant immunochemotherapy. **(A)** Biopsy before neoadjuvant immunochemotherapy (PD-L1, IHC). **(B)** Surgically resected tissue after neoadjuvant immunochemotherapy and surgery (PD-L1, IHC). **(C)** Representative images of the pretreatment biopsy (multiplex immunofluorescence staining, magnification, ×200). **(D)** Representative images of surgically resected tissue (multiplex immunofluorescence staining, magnification, ×200). With this staining technique, visible structures include CD8^+^ T cells (green), CD68^+^ macrophages (cyan), CD57^+^ cells (red), PD-1^+^ cells (magenta), and PD-L1^+^ cells (orange). **(E, F)** Quantitative multiplex immunohistochemistry results of pretreatment and posttreatment samples in the tumor and stroma regions.

**Figure 4:**
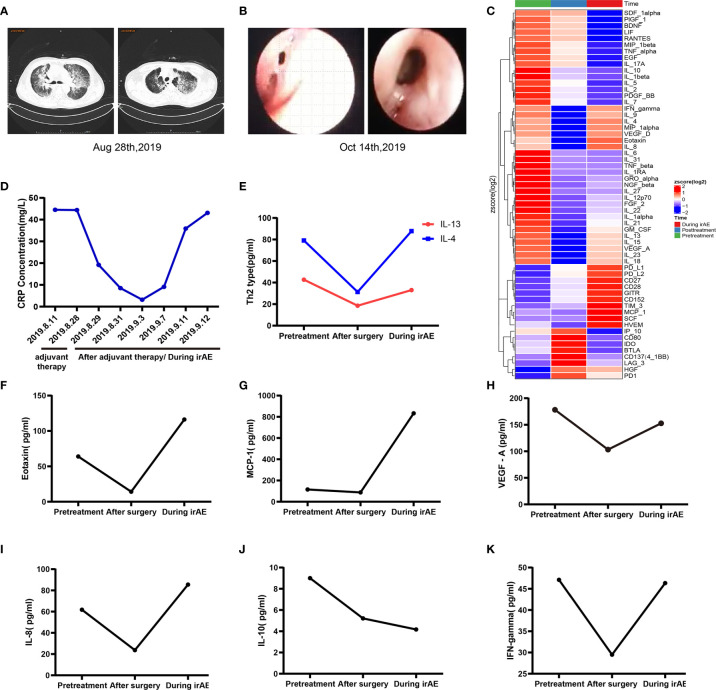
Changes in related indexes during adjuvant immunochemotherapy and irAEs. **(A)** Pulmonary CTA indicated multiple cords, plaques, and flocculent high-density shadows in both lungs, bilateral pleural effusion, and a clinical diagnosis of immune-associated pneumonia (CTCAE level 4). **(B)** Bronchoscopy revealed a fistula at the anastomosis of the right bronchus and the formation of a bloody abscess cavity. **(C)** Multiplexed bead immunoassay results during the entire treatment period. **(D)** Representative CRP levels during adjuvant immunochemotherapy and irAEs. **(E–K)** Representative multiplexed bead immunoassay results during the entire treatment period.

In April 2019, a 62-year-old male patient was admitted to the hospital for paroxysmal cough for 3 months. The clinical course and examination are shown in **Figure 1**. The patient was an active smoker for 40 years. He was diagnosed with emphysema and pulmonary bullous disease 10 years ago. He was diagnosed with hypertension 5 years ago and received amlodipine treatment through oral administration (dose, 5 mg/per day). During the clinical evaluation, a stage IIIA squamous cell lung carcinoma (cT2bN2M0) was diagnosed on the basis of a pathological biopsy, chest-enhanced CT, and positron-emission tomography/computed tomography (PET/CT). The primary tumor was assessed by hematoxylin and eosin staining. Tumor cells accounted for 90% of the cells in the pretreatment tumor biopsy (**Figure 2A**). Then, the patient received nivolumab combined with albumin-bound paclitaxel and carboplatin therapy for three cycles, as follows: nivolumab (200 mg, on day 1, every 2 weeks, 3 cycles), albumin paclitaxel (200 mg, on days 1 and 8, every 3 weeks, 3 cycles), and carboplatin (300 mg, on days 2 and 3, every 3 weeks, 3 cycles). Surgery was performed 29 days after the first day of the third treatment cycle. The patient was started on anti-cancer treatment concomitant with amlodipine treatment (dose, 5 mg/per day). After neoadjuvant PD-1 blockade and chemotherapy, radiological images showed that the tumor was significantly reduced (**Figure 2A**). Serum tumor marker levels (CEA and CA125) were decreased compared with pretreatment levels (**Figure 2B**). Pathological assessment showed that the residual viable tumor cells accounted for 60% of the resection sample (**Figure 2C**). The patient exhibited 30% pathological regression of the primary tumor. We evaluated PD-L1 expression, the infiltrating immune cells, and the mutation profile in the pretreatment tumor biopsy and the post-treatment resection specimen. In the pretreatment tumor biopsy, the immunohistochemical results showed that tumor cell PD-L1 expression was negative (**Figure 3A**). Multiplex immunofluorescence analyses showed that a few CD8^+^ T cells, CD8^+^ PD-1^+^ T cells, CD57^+^ NK cells, CD68^+^ macrophages, and CD68^+^ PD-L1^+^ macrophages infiltrated the tumor (**Figure 3B**). Next-generation sequencing (NGS) showed wild-type *EGFR, ALK, ROS, NTRK*, and *STK11* genes and mutant *TP53* (p.E68*, variant allele frequency [VAF], 11.21%). The microsatellite state was microsatellite stable (MSS), and tumor mutational burden (TMB) was low (TMB value of the tumor biopsy was 5.15 mut/Mb. TMB cutoff is 12 mut/Mb). In the post-treatment resection specimen, PD-L1 immunohistochemistry (IHC) assays showed that PD-L1 expression was 50% in the resected tumor (**Figure 3C**). The mIHC results revealed more CD8^+^ and CD8^+^ PD-1^+^ T cell infiltration posttreatment relative to pretreatment (**Figures 3D–F**). After surgery, the patient received nivolumab (200 mg, on day 1, every 2 weeks, two cycles) combined with albumin-bound paclitaxel (200 mg, on days 1 and 8, every 3 weeks, two cycles) and carboplatin (300 mg, on days 2 and 3, every 3 weeks, two cycles) therapy beginning on July 29th. During adjuvant therapy, immune-related lung injury (grade 4) occurred (**Figure 4A**). The chest CT showed multiple cords, plaques, and flocculent high-density shadows in both lungs, as well as bilateral pleural effusion. EB and cytomegalovirus virus tests were negative. Procalcitonin and fungal d-glucan were normal. These findings did not support any pulmonary infection. Next, the patient received methylprednisolone, anti-infectives, respiratory support, and support therapy. On October 14th, bronchoscopy showed hemorrhagic vomica in the bronchial anastomosis (**Figure 4B**). Unfortunately, the patient died on October 15th. Serum cytokine changes during the neoadjuvant phase and adjuvant PD-1 blockade combined with chemotherapy therapy were detected by multiplexed bead immunoassays (Genecast, Wuxi, China) (**Figure 4C**). Serum levels of C-reactive protein (CRP), Th2 cytokines (IL-13 and IL-4), eotaxin, VEGF-A, IL-8, and IFN-gamma were decreased after neoadjuvant immunochemotherapy and increased during adjuvant immunochemotherapy. The serum levels of MCP-1 did not change significantly during neoadjuvant therapy and increased during adjuvant therapy. The serum level of IL-10 was consistently decreased during neoadjuvant and adjuvant immunochemotherapy (**Figures 4D–K**).

## Discussion and Conclusions

The following observations during the clinical course of the patient should be noted: the pathological regression in response to neoadjuvant immunochemotherapy and irAEs occurred during adjuvant immunochemotherapy. Based on the current case report and the literature, possible mechanisms of the observed clinical phenomena may involve the following. First, after neoadjuvant PD-1 blockade combined with chemotherapy therapy, the tumor immune environment was reshaped, as reflected by increased immune cell infiltration. Chemotherapy can induce the death of immunogenic cells, release tumor-associated neoantigens, and trigger immune activation (11–13). After immunochemotherapy, it may promote the release of tumor-specific neoantigens that can activate the immune system, recruit related immune cells, and turn a “cold” tumor into a “hot” tumor. In the NADIM study, it is reported that several lymphocytes, including CD3^+^ and CD8^+^T cells, were significantly reduced after chemoimmunotherapy (9). The authors inferred that this may be associated with the reduced or completely eliminated tumor mass. In our case, we found that there was an increase of PD-L1 expression, as well as CD8^+^ and CD8^+^/PD1^+^ T cells in the post-neoadjuvant specimen, compared with baseline. We inferred that the difference among histology findings reported in the NADIM study and our case report may be as follows. In the NADIM study, a high proportion of tumors achieved a major or complete pathological response (9). They also observed a significant reduction in CD8^+^ T cells in tumors with complete pathological response. In our case, although the patient had pathological regression, the tumor did not achieve a major or complete pathological response. Actually, tissue heterogeneity was the confounding factor when comparing the difference between the biopsy tissue and surgical specimen. A good prognosis was observed in wild-type *STK11/EGFR* lung adenocarcinoma patients with mutant *TP53* who received anti-PD-1 therapy (14). In this case, the patient harbored wild-type *STK11/EGFR* and mutant *TP53*. The frequency of *TP53* mutation in the pretreatment biopsy (65.79%) and surgically resected tissue (11.21%) suggests that *TP53* mutation was the main driving mutation in the patient, which is consistent with the clinical outcome. This finding suggests that *TP53* mutation might be a biomarker for neoadjuvant PD-1 blockade combined with chemotherapy. Second, the mechanisms underlying the irAEs may be elucidated on the basis of changes in serum cytokine levels during immunochemotherapy. In this case, the serum levels of Th2 cytokines (IL-13 and IL-4), eotaxin, VEGF-A, IL-8, and MCP-1 were increased during adjuvant PD-1 blockade combined with chemotherapy. A previous study reported that a lung adenocarcinoma patient was diagnosed with acute eosinophilic pneumonia after PD-1 blockade (nivolumab) therapy (15). Acute eosinophilic pneumonia is a Th2 inflammation-related lung disease characterized by eosinophilic infiltration of the lung parenchyma (16, 17). Several studies have indicated that PD-L2 regulates the production of Th2 cytokines and airway hypersensitivity (18–20). After PD-1 blockade therapy, the PD-1-PD-L2 interaction may be blocked, and Th2-type inflammation may be induced. Th2 cytokines, eotaxin, VEGF-A, MCP-1, and IL-8 play essential roles in the recruitment and activation of eosinophils in lung disease. The IL-10 cytokine can repress proinflammatory responses and has indispensable functions for inhibiting unnecessary inflammation. In this case, the serum level of IL-10 consistently decreased throughout the clinical course. Therefore, the dysfunction in IL-10 may also be involved in inflammation-related lung disease. Since the biopsy was not performed during the adjuvant immunochemotherapy, it lacks pathological evidence supporting the accurate diagnosis of immune-related pneumonitis in this case. We inferred that the risk factors for enhanced toxicity of adjuvant immunochemotherapy may be as follows. The surgery was performed early after neoadjuvant immunochemotherapy. In the NADIM study, surgery was planned 42 to 49 days after the first day of the third treatment cycle. In this case, the surgery was performed 29 days after the first day of the third treatment cycle of neoadjuvant chemo/immunotherapy. In our case, the surgery may have been performed too early. The optimal timing of surgery may be at least 3 or 4 weeks or longer after the neoadjuvant chemo-immunotherapy, as inferred in the NADIM study. The association between the timing of surgery and incidence of irAEs is worthy of investigation in the future. The optimal timing of surgery may increase the benefit from neoadjuvant chemo-immunotherapy. In addition, the patient was diagnosed with emphysema and pulmonary bullous ten years ago. This may increase the risk of lung toxicity. Furthermore, we observed that the bronchoscopy showed hemorrhagic vomica in the bronchial anastomosis. This may be a sign of anastomosis leakage. Based on these findings, it was inferred that the patient’s cause of death may include several factors, such as anastomosis leakage or/and irAEs.

In summary, the present case describes a squamous cell lung carcinoma patient who exhibited pathological regression in response to neoadjuvant PD-1 blockade combined with chemotherapy therapy, which reshaped the tumor immune environment. The case indicated that PD-L1 negative, *EGFR*/*STK11* wild-type, and *TP53* mutant SQCC patients with a few immune cells infiltrated would likely respond to neoadjuvant immunochemotherapy. Unfortunately, the patient died from anastomosis leakage or/and irAEs. This study may improve our understanding of how PD-1 blockade acts as a double-edged sword.

## Data Availability Statement

The raw data supporting the conclusions of this article will be made available by the authors, without undue reservation.

## Ethics Statement

The studies involving human participants were reviewed and approved by Ethics Committee of People’s Liberation Army (PLA) 81 Hospital (Nanjing, China). The patients/participants provided their written informed consent to participate in this study. Written informed consent was obtained from the individual(s) for the publication of any potentially identifiable images or data included in this article.

## Author Contributions

Conception and design: LH, YH, JH, YL, LW, and DongW. Administrative support: YL and XG. Provision of study materials or patients: LH, JH, and LW. Collection and assembly of data: YH and YL. Data analysis and interpretation: YH, YL, LH, JH, BM, HC, and DiW. Manuscript writing: LH, YH, JH, and YL. All authors contributed to the article and approved the submitted version.

## Conflict of Interest

Authors YL, HC, XG and DiW were employed by Genecast Biotechnology Co., Ltd.

The remaining authors declare that the research was conducted in the absence of any commercial or financial relationships that could be construed as a potential conflict of interest.

## Abbreviations

NSCLC, non-small cell lung cancer; MPR, major pathological response; pCR, pathologic complete response; irAEs, immune-related adverse events; PET/CT, positron-emission tomography/computed tomography; NGS, next-generation sequencing; CRP, C-reactive protein; MSS, microsatellite stable; TMB, tumor mutational burden.
